# The top 1%: quantifying the unequal distribution of malaria in Brazil

**DOI:** 10.1186/s12936-021-03614-4

**Published:** 2021-02-12

**Authors:** Raquel Lana, Narimane Nekkab, Andre M. Siqueira, Cassio Peterka, Paola Marchesini, Marcus Lacerda, Ivo Mueller, Michael White, Daniel Villela

**Affiliations:** 1 Scientific Computing Programme, Fundação Oswaldo Cruz, Rio de Janeiro, 21040-360 Brazil; 2 Malaria: Parasites and Hosts, Department of Parasites and Insect Vectors, Institut Pasteur, Paris, France; 3 Instituto Nacional de Infectologia Evandro Chagas, Fundação Oswaldo Cruz, Rio de Janeiro, 21040-360 Brazil; 4 Fundação de Medicina Tropical Dr. Heitor Vieira Dourado, Manaus, AM Brasil; 5 Programa de Pós-Graduação Em Medicina Tropical, Universidade Do Estado Do Amazonas, Manaus, AM Brasil; 6 Programa Nacional de Controle da Malária, Ministério da Saúde, Brasília, DF Brasil; 7 Department of Transmissible Diseases Surveillance, Ministry of Health, Brasília, Brazil; 8 Instituto de Pesquisas Leônidas and Maria Deane, Fundação Oswaldo Cruz, Manaus, AM Brasil; 9 Division of Population Health and Immunity, Walter and Eliza Hall Institute, Melbourne, Australia

**Keywords:** Malaria, *Plasmodium vivax*, *Plasmodium falciparum*, Epidemiology

## Abstract

**Background:**

As malaria endemic countries strive towards elimination, intensified spatial heterogeneities of local transmission could undermine the effectiveness of traditional intervention policy.

**Methods:**

The dynamic nature of large-scale and long-term malaria heterogeneity across Brazilian Amazon basin were explored by (1) exploratory analysis of Brazil’s rich clinical malaria reporting database from 2004 to 2018, and (2) adapting Gini coefficient to study the distribution of malaria cases in the region.

**Results:**

As transmission declined, heterogeneity increased with cases clustering into smaller subpopulations across the territory. In 2004, the 1% of health units with the greatest number of cases accounted for 46% of all reported *Plasmodium vivax* cases, whereas in 2018 52% of *P. vivax* cases occurred in the top 1% of health units. *Plasmodium falciparum* had lower levels of transmission than *P. vivax*, and also had greater levels of heterogeneity with 75% of cases occurring in the top 1% of health units. Age and gender stratification of cases revealed peri-domestic and occupational exposure settings that remained relatively stable.

**Conclusion:**

The pathway to decreasing incidence is characterized by higher proportions of cases in males, in adults, due to importation, and caused by *P. vivax*. Characterization of spatio-temporal heterogeneity and risk groups can aid stratification for improved malaria control towards elimination with increased heterogeneity potentially allowing for more efficient and cost-effective targeting. Although distinct epidemiological phenomena were clearly observed as malaria transmission declines, the authors argue that there is no canonical path to malaria elimination and a more targeted and dynamic surveillance will be needed if Brazil decides to adopt the elimination target.

## Background

As countries accelerate towards malaria elimination, reduction in malaria transmission is highly variable leading to significant heterogeneities in residual transmission across their territories [1–4]. In such circumstance, a ‘one size fits all’ approach to public policy and intervention may no longer be cost-effective and may slow progress towards the final goal of local elimination of transmission. Understanding both the dynamic nature of these heterogeneities and developing systems to efficiently identify and characterize residual transmission areas is essential to developing locally adapted, sub-national malaria elimination policies and intervention packages.

Brazil has seen long-term trends of nationwide reductions in notified *Plasmodium vivax* and *Plasmodium falciparum* cases since 2000, and a retreat of malaria into the Amazon Basin in the north of the country [5]. These epidemiological trends have been dependent on socio-economic development, and sustained effective case management, largely based on intensive passive case detection (PCD) [3]. In Brazil, treatment is provided publicly and free of charge in government-run Health Units, including hospitals and mobile healthcare workers in both urban and rural communities [6]. From these Health Units, data on treated cases are digitally recorded in the Malaria Epidemiological Surveillance Information System (SIVEP) database. Containing digitized data on almost 6 million malaria cases with individual level covariates, the Brazilian SIVEP database is one of the most detailed health systems databases ever assembled in a malaria endemic country, covering the entire endemic region and resulting in virtually no underreporting [7, 8]. The collected data span a crucial period, as many regions of Brazil are nearing and some even achieving local malaria elimination, providing examples of what malaria elimination looks like from a health system perspective.

In addition to the long-term, large-scale trends in malaria epidemiology, there is considerable variation in the numbers of notified cases between regions and over time. Factors contributing to spatio-temporal heterogeneity include deforestation, changes in land use and economic activity, ecological suitability of mosquitoes (most notably of *Anopheles darlingi*), climate variations, internal and international migration, and political crises in neighbouring countries [9–11]. Historically, the Brazilian malaria landscape was largely shaped by what has been referred to as ‘frontier malaria’ where new infections were driven by non-immune populations settling for economic and political purposes [3, 12–14]. More recently, although malaria is in an epidemiological transition towards elimination in some regions, it has maintained itself as an endemic disease with sustained transmission over many years in many regions [2, 3]. Despite this heterogeneity, policies on surveillance, diagnosis, and antimalarial treatment are exactly the same for the whole country. Major guidelines are applied to all states and municipalities, each being responsible for executing actions with little flexibility or customization regarding specific scenarios [9].

Micro-epidemiological studies provide an in-depth local understanding of malaria transmission, and are crucial for designing stratified locally adapted intervention strategies [15]. Data from micro-epidemiological settings can be aggregated to construct a macro-epidemiological picture of malaria at varying regional levels, such as within Brazilian states, or across the entire nation of Brazil. Understanding how thousands of such micro-epidemiological patterns combine to produce the macro-epidemiology of malaria in Brazil is a challenging problem.

Data aggregation across multiple levels is a more general problem encountered in many fields. In economics, there are many statistical tools for quantifying the relationship between micro-economic and macro-economic phenomena. An important example is the Gini coefficient, an index which quantifies wealth inequality between individuals on a national level. Recent advances in health systems databases allow for conceptual parallels between economic and epidemiological phenomena to be exploited [16]. For example, statistical tools originally designed for the analysis of wealth inequality can be repurposed to provide insight into malaria heterogeneity.

Improved understanding of heterogeneity across multiple spatial scales will better inform stratification of malaria risk zones allowing for more efficient targeting of interventions. In Brazil, the National Malaria Control Programme (NMCP) already pursues a targeted approach with vector control interventions being distributed to high risk areas [10]. In this macro-epidemiological analysis of Brazilian SIVEP-malaria health system data, it is demonstrated how a Gini index applied to malaria cases over different spatial units (state, municipality, and others) describes heterogeneity of malaria transmission in a way that can aid stratification for improved malaria control. This could be a necessary first step in order to move the national strategy from control to malaria elimination.

## Methods

### Data

#### Epidemiological database

Anonymized malaria case notifications were obtained from the Malaria Epidemiological Surveillance Information System (SIVEP) reported between January 2003 and December 2018. SIVEP is specific for the Brazilian Amazon Basin consisting of seven states in the north region plus two state in the northeast: Acre (AC), Amapá (AP), Amazonas (AM), Maranhão (MA), Mato Grosso (MG), Pará (PA), Rondônia (RO), Roraima (RR) and Tocantins (TO).

Data on notified malaria cases from the first year of SIVEP, 2003, were excluded because of uncertainties related to the roll out of the system. Cases were organized by malaria type: *P. vivax*, *P. falciparum* and mixed infection. Other types of malaria were excluded. Mixed infections were counted as both *P. vivax* and *P. falciparum*. SIVEP also provides information on whether the notification is a passive case detection, active case detection or cure verification thick smear. The last category was removed from the analysis to avoid multiple counts of single cases. Notifications with inconsistencies in age were excluded.

Extra-Amazonian malaria case notifications were obtained from Information System of Notification Disease (SINAN) from January 2004 to December 2018.

#### Demographic database

The annual population size for each of 5570 municipalities was obtained from the Brazilian Institute of Geography and Statistics (IBGE), based on data from the 2010 census, with modelled population estimates for other years [17]. For ten newly created municipalities between 2003 and 2013, missing population sizes were estimated using spline interpolation.

Since health units were discretized into urban and rural zones but the catchment population was unknown, we inferred the population size by using the 2010 census. For all years, the urban and rural zone populations for each municipality were estimated by inferring from the ratio reported in the census and the estimated population size per year. For each year, the number of active urban and rural health units per municipality were counted from the health unit database and divided the rural and urban municipality population size by the total number of active rural and urban health units.

### Incidence

For each administrative level (the Amazon Basin, states, municipalities, and health units), the incidence was estimated as the number of annual positive exams by the population size per 1000 for each malaria species. These values are an estimate of the true incidence since often cases reported in SIVEP are clinical cases and include relapses. According to NMCP, low transmission areas are classified by an incidence < 10 cases / 1,000 persons, intermediate transmission between 10 to 50 episodes/1,000 inhabitants and high transmission with an incidence > 50 episodes/1,000-inhabitants [18].

It was assumed that health units that reported cases had the same catchment population size if they were located in the same urban or rural zone of the municipality. The type or size of the reporting health unit was not accounted for due to data unavailability.

### Gini coefficient

The Gini coefficient is commonly used as an econometric indicator for wealth inequality. This metric was adapted to assess the distribution of notified malaria cases across the Amazon Basin as a proxy for heterogeneity over space and time. *P. vivax* and *P. falciparum* heterogeneity is measured at the state, municipality, and health unit level. For *N* administrative units, let *P*_*i*_ denote the proportion of the population in unit *i*, and *C*_*i*_ denote the proportion of cases in unit *i*. By ranking units in order of decreasing incidence (i.e. by *C*_*i*_/*P*_*i*_), the Gini coefficient can be calculated as

$$G = 2\left( {\sum\limits_{i = 0}^N {\left( {{P_i} - {P_{i - 1}}} \right)\frac{{{C_i} + {C_{i - 1}}}}{2}} } \right) - 1$$

where *P*_*0*_ = 0 and *C*_*0*_ = 0. If cases are randomly distributed such that there is no heterogeneity, then *G* = 0. As heterogeneity increases, and cases are concentrated in smaller subpopulations, then *G* → 1 (its maximum value).

Shapefiles were obtained from the malariaAtlas R package [19]. All plots and analyses were generated using R Software [20].

## Results

### Baseline epidemiology

**Fig. 1:**
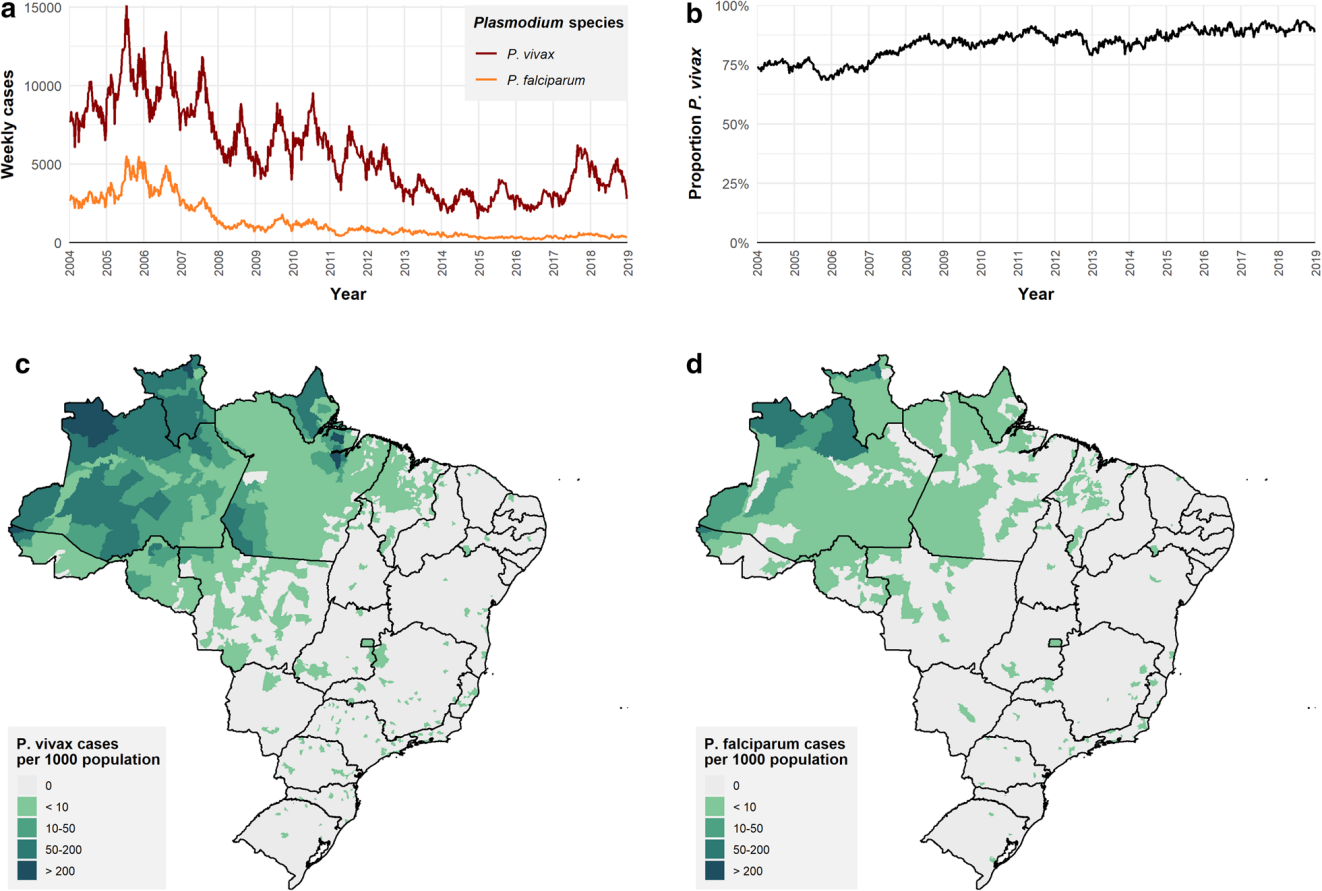
Epidemiology of malaria in Brazil.** a** Weekly *P. vivax* and *P. falciparum* cases. **b** The percentage of notified malaria cases due to *P. vivax*. **c** *P. vivax* annual parasite index (API) by municipality in 2018, measured as notified cases per 1000 population in one year. **d** *P. falciparum* API by municipality in 2018. Mixed infections are counted as both *P. vivax* and *P. falciparum* infections

From 2004 to 2018, 5,496,673 malaria cases were notified in the Brazilian Amazon Basin: 4,443,459 (81%) were *P. vivax*; 990,280 (18%) were *P. falciparum*; 40,829 (0.7%) were *P. vivax* / *P. falciparum* mixed infections; and 22,105 (0.4%) were other malaria species (mainly *Plasmodium malariae*). For Extra-Amazonian malaria, between 2004 and 2018, there were 19,461 notified confirmed cases, among which 11,214 cases (57.6%) were reported as *P. vivax*; 4,152 cases were reported as *P. falciparum* (21.3%); and the remainder were either other species or not reported. There has been a decrease in malaria cases in Brazil following an epidemic in 2005–2006 (Fig. 1a, Additional file 1: Table S1). This nationwide decline obscures important asynchronous patterns such as the malaria epidemic in Pará state between 2009 and 2012 (Additional file 1: Fig. S1, Table S1). Between 2017 and 2018, there was a substantial increase in *P. vivax* and *P. falciparum* cases, with notable increases in the states of Roraima and Amazonas bordering Venezuela (Additional file 1: Table 1, Figs. S1 and S3).

The incidence of malaria was unevenly distributed across the 619 municipalities that reported at least one case in 2018 (Fig. 1, Additional file 1: Fig. S2). Of these municipalities 58% reported fewer than 10 cases in 2018. The highest *P. vivax* transmission in 2018 was in Oeiras do Pará municipality with over 15,000 cases and an API of 468. The highest *P. falciparum* transmission in 2018 was in São Gabriel da Cachoeira, Amazonas with 4,800 cases and an API of 109.

Most malaria transmission events occur in the Amazon basin. The distribution of API over the country clearly shows much higher incidence in the Amazon basin (Fig. 1), whereas API in municipalities out of the Amazon basin had API values smaller than 1 per 1000 inhabitants.

### Age and gender distribution of cases reflect varying modes of transmission

**Fig. 2:**
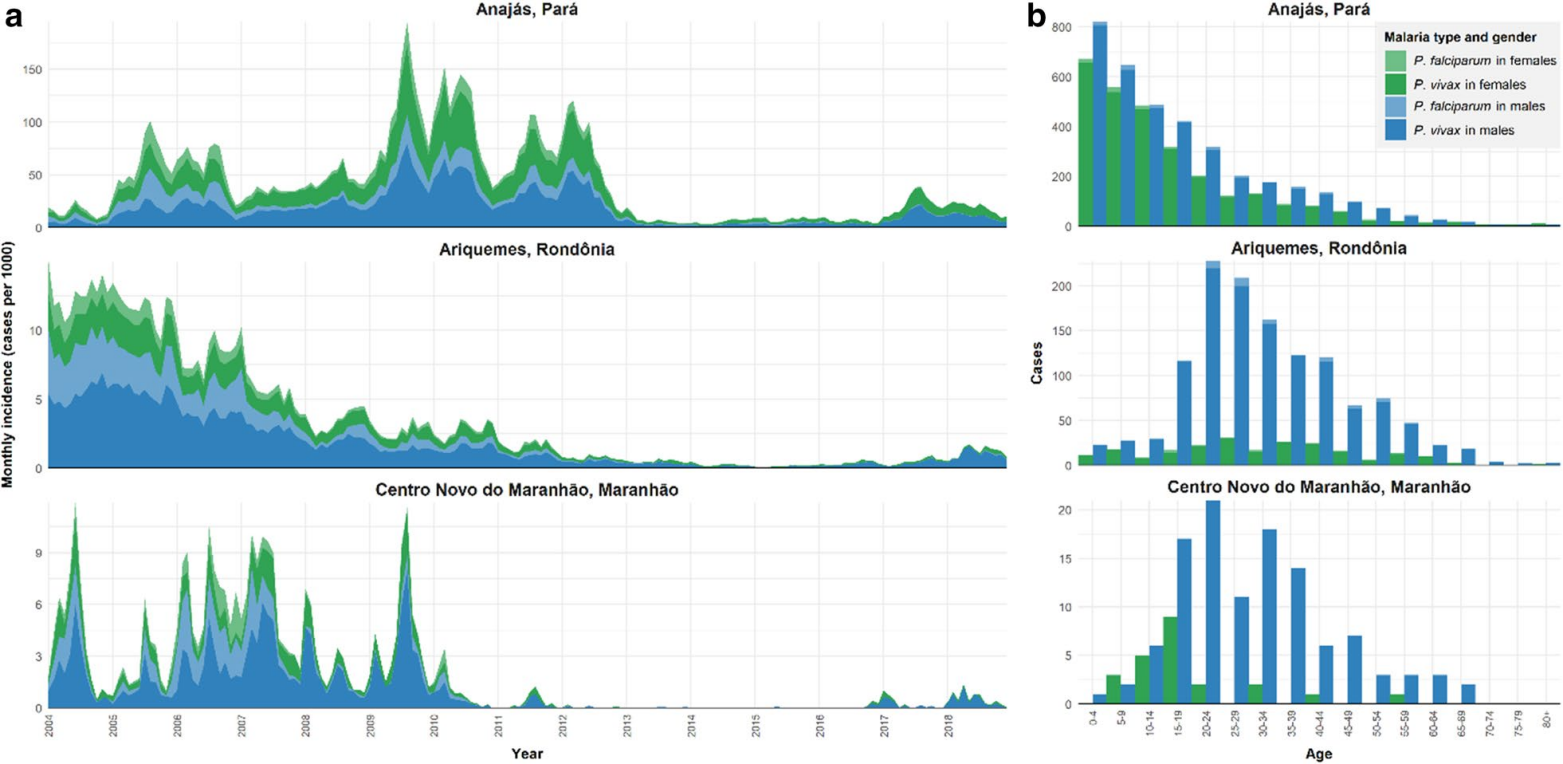
Age and gender stratification of malaria cases. **a** Time series of malaria cases stratified by gender and malaria species for a selection of municipalities. **b** Notified *P. vivax* cases stratified by age in selected municipalities in 2018

Across Brazil in 2018, more cases were notified in males (60.5%) than in females (39.5%). Stratification of time series by gender reveals considerable diversity in local epidemiology (Fig. 2a), when analysing different patterns of age/gender incidence between low and high-transmission municipalities. A few selected examples from the exploratory data analysis are considered in more detail. In high transmission Anajás there are comparable numbers of cases in both males and females. In low transmission Ariquemes and in near elimination Centro Novo do Maranhão there are substantially more cases in males than in females. In 2018, the remaining cases are almost exclusively due to *P. vivax* in males: 85% in Ariquemes and 83% in Centro Novo do Maranhão.

Stratification of cases by age reveals additional distinct patterns within municipalities (Fig. 2b) across a range of transmission settings. In many high transmission municipalities such as Anajás, a large proportion of cases occur in children (59%). In contrast, in many low transmission settings such as Ariquemes and Centro Novo do Maranhão, the majority of cases were reported in working age (16–65 years) males.

Malaria cases can be stratified by age, gender and species in all states in the Amazon Basin, allowing for identification of the pre-dominant exposure routes (Additional file 1: Fig. S4). In Acre, Amazonas and Pará states, the high proportion of cases in children under 16 years indicates peri-domestic exposure. For example, Acre contains three municipalities (Cruzeiro do Sul, Mâncio Lima and Rodrigues Alves), which constitute one of the most intense pockets of malaria endemicity in the Americas [21]. In 2018, these municipalities reported incidence of 149, 413, and 172 cases per 1000 inhabitants, respectively. This perspective from the SIVEP database is supported by local epidemiological data from Mâncio Lima where fishponds built near households have been reported to drive peri-domestic transmission [22]. In Amapá, Maranhão, Mato Grosso, Rondônia and Roraima states, peaks of cases in working-age males (16 to 65 years old) indicate occupational exposure. In all states, the age and gender distribution of cases was relatively stable over time suggesting very little change in the underlying exposure patterns (Additional file 1: Fig. S4).

### Spatial heterogeneity in malaria cases

**Fig. 3:**
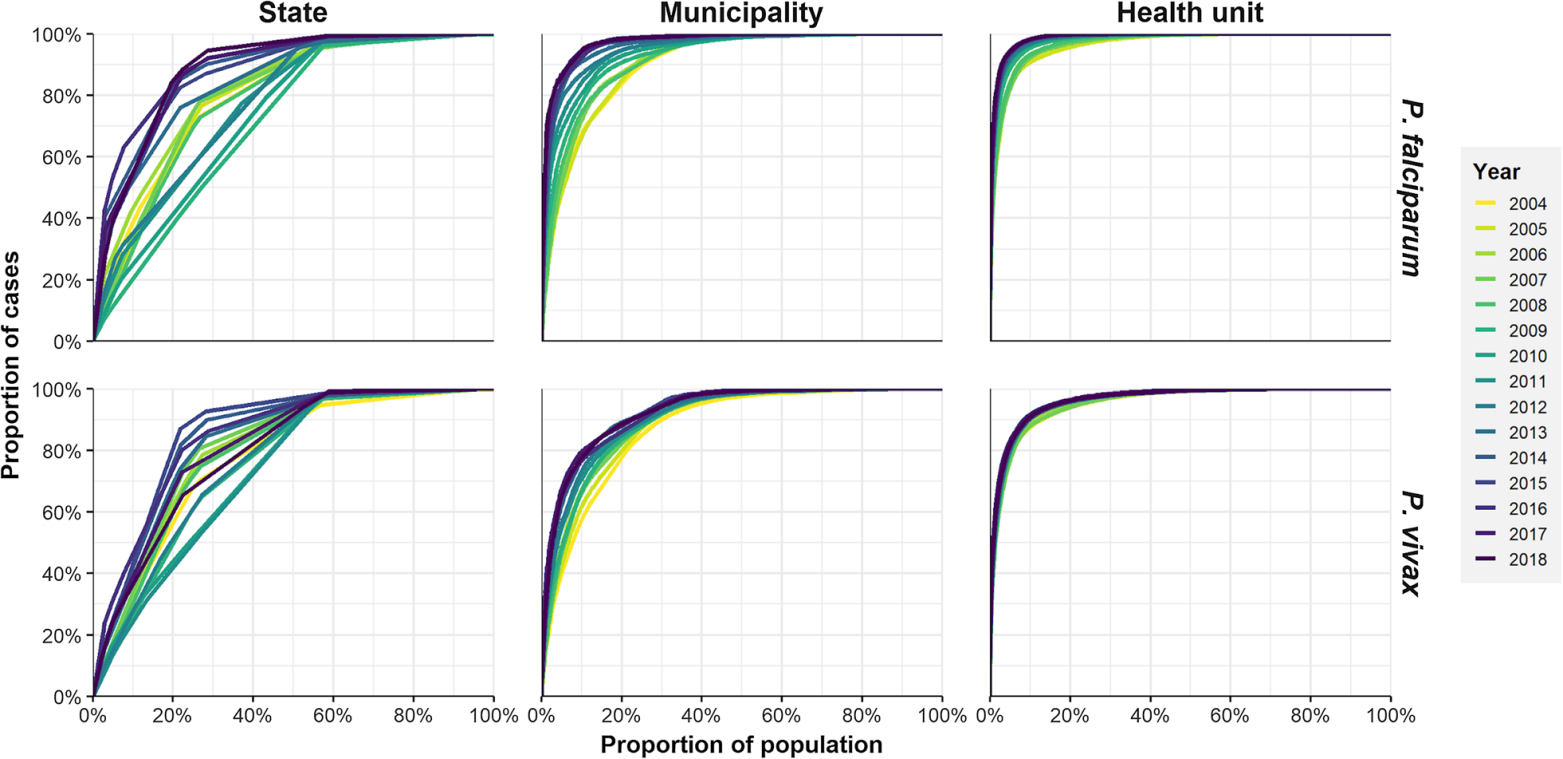
Cumulative distribution of malaria cases at varying spatial scales in the Amazon Basin. Three administrative levels (state, municipality, health unit) are considered, corresponding to different spatial resolution. Administrative units are ranked according to *P. vivax* or *P. falciparum* transmission intensity. For example, 80% of *P. falciparum* cases in 2004 could be found in municipalities containing 20% of the population in the Amazon Basin

Measuring malaria transmission via notified cases can be done on different administrative levels (corresponding to spatial area), according to measured case numbers and denominator population. For example, at the state level, Acre state had the highest *P. vivax* incidence in 2018; whereas the municipality with the highest *P. vivax* incidence was Oeiras do Pará in Pará state. The state with the highest number of notified *P. vivax* cases was Amazonas due to its large population. The heterogeneity in transmission intensity of both *P. vivax* and *P. falciparum* is characterized in Fig. 3 for three different administrative levels: state, municipality and health unit.

Heterogeneity in notified malaria cases can be characterized by the cumulative distributions in Fig. 3, with greater area under the curve indicating higher levels of heterogeneity. The area under the curve depends on administrative level—lower levels correspond to higher spatial resolution allowing for more detailed characterization of malaria heterogeneity. Variations were observed over time and between malaria species. For example, in 2004, 80% of *P. vivax* cases were notified in 99 municipalities containing 21% of the population. In 2018, higher heterogeneity was observed with 80% of *P. vivax* cases notified in 75 municipalities containing 12.5% of the population. At the lower administrative level of health units, 80% of *P. vivax* cases were notified in 573 health units representing 4.5% of the population in 2018 compared to 839 health units in 2004. In relation to *P. falciparum* cases, heterogeneity was even more pronounced over time and space with 80% of notified cases reported in 25 municipalities representing 3.1% of the population in 2018.

### Increasing heterogeneity on the path to elimination

Heterogeneity in notified malaria cases between administrative units can be quantified using the Gini coefficient—a tool borrowed from econometrics. The calculation of the Gini coefficient is based on the area under the cumulative distribution curves in Fig. 3, with higher values indicating greater heterogeneity.

**Fig. 4:**
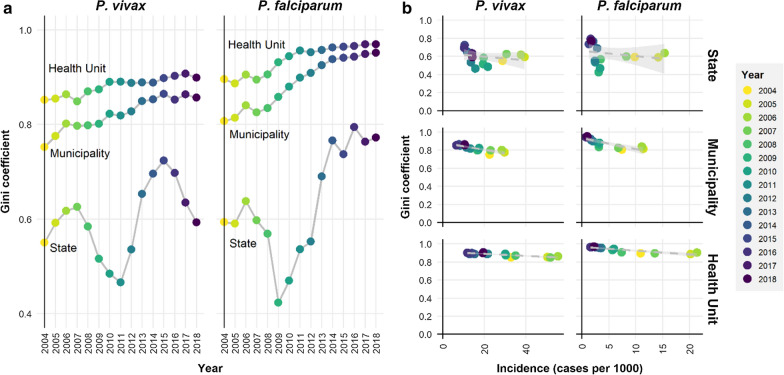
Analysis of spatio-temporal heterogeneity with the Gini coefficient.** a** Spatial heterogeneity as measured by the Gini coefficient has been increasing over time for both *P. vivax* and *P. falciparum* cases at the administrative levels considered. **b** Lower malaria incidence is associated with increased Gini coefficient

The calculated Gini coefficient depends on the administrative level considered (Fig. 4a). Stratification at lower administrative levels (corresponding to smaller population sizes) provides higher spatial resolution, allowing more heterogeneity in malaria cases to be quantified (Additional file 1: Fig S6). At all administrative levels considered, heterogeneity was observed to increase over time (Fig. 4a).

At the state level the Gini coefficient fluctuated over the years, but incidence also varied during this period. At the municipality and health unit level, the coefficient was more stable. The increase in "Gini coefficient" is steady in recent years for *P. falciparum*. In the case of *P. vivax* infections, there is some recent decrease which corresponds to recent increase of *P. vivax* infections. Between the major epidemic in 2006 and 2016, *P. vivax* API fell from 28.5 to 6.5 and *P. falciparum* API fell from 11.1 to 0.8 (Additional file 1: Table 1). During this period of decreasing malaria, the Gini coefficient increased across all administrative levels. At the municipality and health unit levels, there was an approximately linear relationship between Gini coefficient and malaria transmission. Following the rebound of malaria after the lowest transmission period in 2016, the number of cases and the incidence per 1000 of both species increased, and subsequently we observed lower Gini coefficients in 2018.

Heterogeneity was observed to vary over space and time with changing transmission intensity (Fig. 4b). As *P. vivax* and *P. falciparum* transmission decreased, heterogeneity increased with cases being clustered into smaller subpopulations over time across all levels, although this effect may be more subtle depending on levels.

In 2004, the 1% of health units with the greatest number of cases accounted for 46% of all reported *P. vivax* cases (Additional file 1: Fig. S5). Heterogeneity, as measured by this metric, has increased notably over time so that in 2018, 52% of *P. vivax* cases occurred in the top 1% of health units. Over this time period *P. falciparum* transmission was notably lower than *P. vivax* transmission, with more extreme patterns of heterogeneity, with 51% of *P. falciparum* cases reported in the top 1% of health units in 2004, rising to 75% of *P. falciparum* cases in the top 1% of health units in 2018.

### Characterizing the decline of malaria cases

**Fig. 5:**
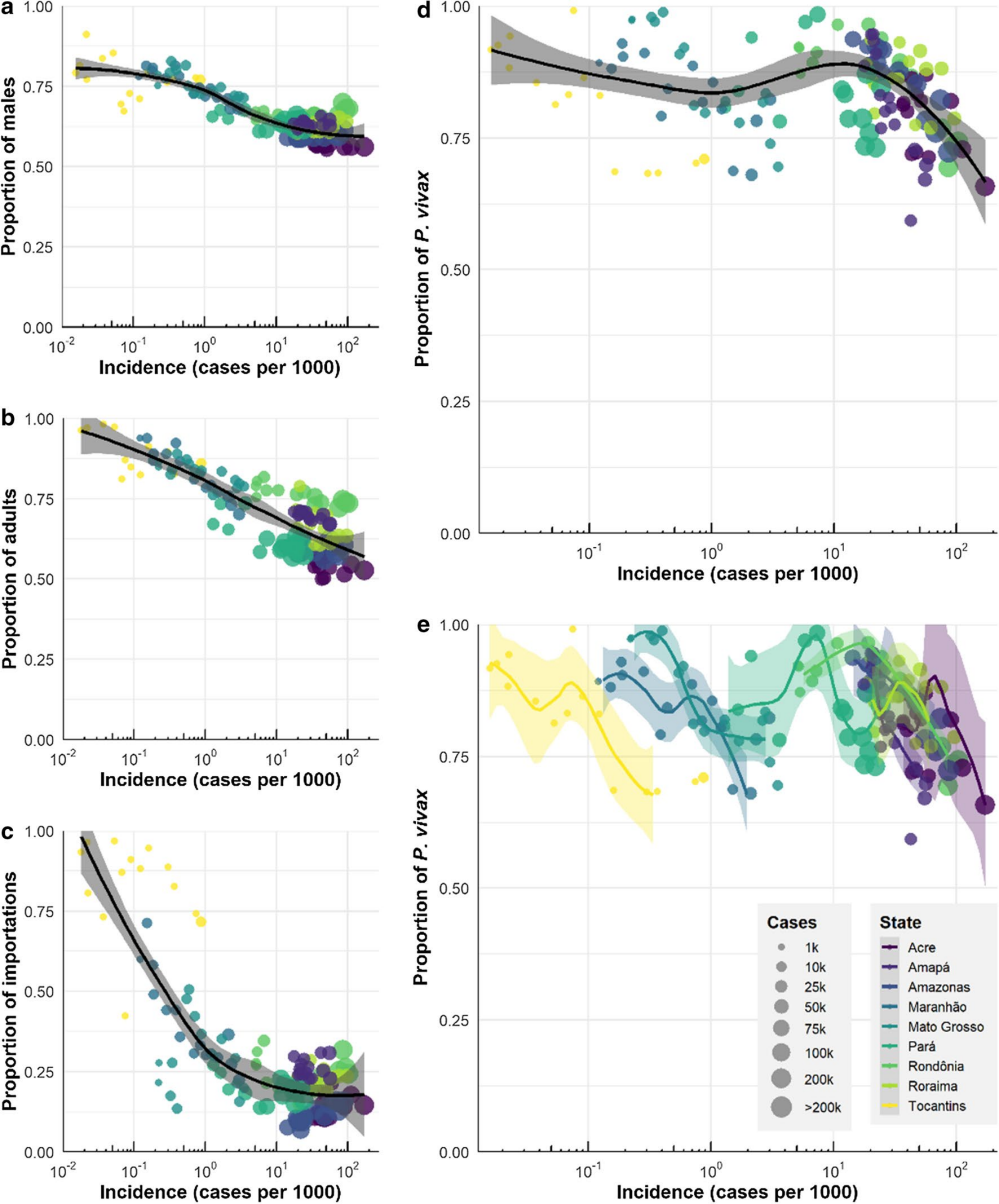
Characterizing the path to elimination.** a** The proportion of notified malaria cases in males increases in lower transmission settings. **b** The proportion of notified malaria cases in adults (> 16 years) increases in lower transmission settings. **c** The proportion of notified malaria cases due to importation increases in lower transmission settings. **d** Across the Amazon Basin, the majority of notified malaria cases are due to *P. vivax*. **e** When analysed on the state level, an increasing proportion of *P. vivax* cases is seen in lower malaria transmission settings. Each point represents one observation per state from 2004 to 2018. The loess curve is estimated using data from 2006 onwards and observations with at least 20 annual cases reported (**a**–**d**: for all points; **e**: by state)

The decline of malaria in the Amazon Basin has been a long-term epidemiological trend since 1960, subject to short-term fluctuations. This retreat of malaria into the western part of the Amazon Basin has been well characterized previously [9, 10], and is captured in the SIVEP database [18], with low numbers of notified cases in the states of Mato Grosso, Maranhão, and Tocantins (Additional file 1: Fig. S4). As regions progress towards elimination, a distinctive path is followed, with the remaining malaria cases more likely to be in adult males, imported, and caused by *P. vivax* (Fig. 5).

For malaria cases notified in high transmission settings, on average 62% were in males, 63% were in adults, 20% were imported, and 77% were due to *P. vivax*. In these high transmission settings, it is expected to observe cases more homogeneously distributed across the population in terms of age and gender with mainly local transmission events. In lower transmission settings, the proportions increased to 74% in males, 82% in adults, 45% imported, and 85% due to *P. vivax*. The most pronounced feature change was the proportion of importations when the incidence dropped to lower than 1 case per 1000 population (Fig. 5c).

Both *P. vivax* and *P. falciparum* cases have been decreasing over time (Fig. 1a). The more rapid decrease in *P. falciparum* cases has caused the proportion of malaria cases due to *P. vivax* to increase over time, from 75% in 2004 to 92% in 2018. There was not a clear association between malaria incidence and the proportion of cases due to *P. vivax* (Fig. 5d). However, when stratified by state, there is an increasing proportion of *P. vivax* cases with declining transmission within states (Fig. 5e).

## Discussion

The majority of cases of malaria in Brazil occur in the Amazon basin. Although there is malaria transmission in some sylvatic areas along the coast of Brazil, most transmission events notified in areas other than the Amazon basin originate from the states in the Northern region of the country. Therefore, any efforts to eliminate malaria depend on the incidence in the Amazon region.

Stratification of at-risk populations at lower administrative levels reveals increasing heterogeneity in the distribution of malaria cases throughout the Brazilian Amazon. This is consistent with malaria having a fractal nature within the levels considered, with evermore variation observed at lower spatial resolution [23]. A limitation of our analysis is that the lowest level with accurate data on cases and denominator populations was the municipality. Although inference was possible for health units within municipalities, this required estimation of catchment populations. However, two lines of evidence indicate that additional heterogeneity would be observed with finer population stratification at lower spatial scales. Firstly, demographic stratification by age and gender reveals substantial heterogeneity. Secondly, micro-epidemiological analyses of data from cross-sectional and longitudinal cohorts in Brazils with geo-located households reveals considerable spatial clustering [24].

Borrowing from econometrics, the Gini coefficient was utilized to demonstrate that heterogeneity in the distribution of malaria cases is increasing over time (Fig. 4a). In particular, this is closely associated with decreasing incidence of malaria cases (Fig. 4b). These results suggest that the spatial distribution of malaria cases across populations was dependent on changing transmission intensity and the size of the population at risk. This pattern of increasing heterogeneity with declining transmission has also been demonstrated on a micro-epidemiological level, with a higher clustering of infectious mosquitoes in lower transmission settings [16].

Stratification of cases by age and gender reveals patterns consistent with two key archetypes of malaria transmission settings in Brazil: peri-domestic and occupational exposure. Peri-domestic transmission is characterized by exposure to infectious mosquitoes in and around peoples’ households. In these settings, age and gender stratification typically reveal cases across all age groups, in both males and females. In particular, the distribution of cases represents the underlying demography of the population. Municipalities with high API are often, but not always, characterized by peri-domestic transmission, as cases are notified from all segments of the population. In agreement with data from sub-Saharan Africa [25], a peak of cases in children is observed, with this peak shifting towards younger ages in higher transmission settings due to the acquisition of clinical immunity (Additional file 1: Fig. S4) [26]. This allows existing routinely collected data to help target interventions for malaria control. Many peri-domestic settings with cases notified across all age groups in males and females would benefit from targeted vector control, as there is evidence to suggest that transmission is occurring in and around households [10, 27].

Occupational transmission is characterized by exposure to infectious mosquitoes in activities such as mining, agriculture, forestry, and construction [3, 28]. Age and gender stratification typically reveal a concentration of cases in working age males. Occupational exposure settings often have low malaria transmission when assessed across an entire population; however, this may obscure intense and stable transmission in core risk groups. Vector control targeted at households is unlikely to be effective in this settings [29]. Classification of malaria exposure into these two archetypes underestimates the diversity of malaria transmission systems. In particular, it may not reflect differences between urban and rural transmission, or indoor and outdoor based transmission systems.

Systematic analyses of the breakdown of cases across the range of transmission intensities reveals important characteristics of the pathway to elimination. Namely, a higher proportion of cases are seen in males, in adults, due to importation, and due to *P. vivax*. An analysis of data on notified measles cases argued that there is a canonical path to measles elimination, with all regions going through similar epidemiological transitions as they progress towards measles elimination [30]. Although distinct epidemiological phenomena are clearly observed on the path to elimination, the data presented here suggest that there is no canonical path to malaria elimination. Take the example of Tocantins state; although formal elimination has not been declared, the low case numbers and high proportion of importation is consistent with many municipalities having eliminated local malaria transmission in the past two decades. If progress continues over the next two decades, will high transmission municipalities in Acre follow the same trajectory as Tocantins? As Acre is a peri-domestic transmission setting, and Tocantins is an occupational transmission setting, then these regions will not follow the same epidemiological transitions on the way to elimination. More generally, the diversity of malaria transmission systems, suggests that there is no canonical path to malaria elimination, and that each region will need to follow its own path.

The recent history of malaria control in Brazil has mostly been a successful one, with a nationwide reduction of cases, and many municipalities reducing the numbers of notified cases to levels consistent with the elimination of local transmission. This progress has been built on Brazil’s strong case management, with all cases being diagnosed by thick blood smear or rapid diagnostic tests, and treated free of charge at local Health Units. Notably, Brazil is one of few countries that routinely provide radical cure for *P. vivax* via chloroquine, and a total dose regimen of 0.5 mg/kg/day primaquine for 7 days, only excluding children under 6 months and pregnant women. Despite legitimate concerns about this regimen’s adherence and efficacy [31–34], it is playing a key role in the effort for *P. vivax* elimination. The recent approval of tafenoquine for *P. vivax* case management provides important opportunities for malaria control in Brazil. With only a single dose, tafenoquine has been demonstrated to have comparable efficacy to primaquine, although testing for G6PD deficiency will be mandatory due to the risks of treatment-induced haemolysis [35, 36]. The Brazilian NMCP will need to make decisions on whether tafenoquine is implemented universally through a “one-size-fits-all” strategy, or whether roll-out should be targeted at specific regions or risk groups.

The patterns of spatio-temporal heterogeneity demonstrated here have important implications for NMCPs pursuing malaria elimination. The increase in heterogeneity as transmission declines causes the remaining malaria cases to be concentrated in smaller regions or populations, potentially allowing more efficient and cost-effective targeting. For example, rather than implementing standardized interventions across the entire endemic region, targeted policies could be directed at 4.5% of the population or ~ 600 health units, where 80% of cases are known to be reported. However, these remaining pockets sometimes referred to as hotspots can have intense and stable malaria transmission. Sub-microscopic infections undetectable at local clinics may further mask heterogeneous patterns. In such hard-to-eliminate settings, it is crucial to sustain interventions for a long duration and employ multiple strategies that seek to improve in combination radical cure adherence (e.g. single-dose tafenoquine), bed net coverage in remote areas, and diagnostic tools to identify asymptomatic infections.

## Conclusion

The Brazilian SIVEP database provides an important example to be used in malaria control programmes as subsidy for analytical studies to evaluating strategies for malaria elimination. The Gini coefficient proved to be a powerful index to evaluate heterogeneity *i.e*. how few municipalities concentrate the majority of malaria cases. Control programmes might suffer setbacks over time, even unexpectedly. In early 2020, with the emergence of SARS-CoV-2 virus, States in the Amazon basin have been severely affected [37], which can impact on local malaria control due to shortage of human resources, diverted attention in controlling transmission, less capacity in health systems, and loss of funding [38]. Results here show that, despite an unequal concentration of cases, efforts need to be sustained over time in order to continue on the path to malaria elimination.

## Supplementary Information

**Additional file 1: Table S1.** Notified cases and incidence per 1000 population per year, malaria type, highest transmission state of the Amazon Basin. **Figure S1.** Monthly *P. vivax* and *P. falciparum* cases. **Figure S2.** *P. vivax* and *P. falciparum* 2018 cases stratified by municipality. **Figure S3.** States reporting cases imported from Venezuela. **Figure S4.** Proportion of *P. vivax* and *P. falciparum* cases by age group per year in each State stratified by gender. **Figure S5.** Proportion of cases in the top 1% of municipalities and health units. **Figure S6.** Gini coefficient and population size.

## Data Availability

Epidemiological data of malaria cases in Brazil requires a request for SIVEP data to the Ministry of Health in Brazil.

## References

[1] PAHO (2017). Report on the situation of malaria in the Americas 2000–2015.

[2] Braz RM, Barcellos C (2018). [Analysis of the process of malaria transmission elimination with a spatial approach to incidence variation in the Brazilian Amazon, 2016] (in Portuguese). Epidemiol Serv Saude.

[3] Souza PF, Xavier DR, Suarez Mutis MC, da Mota JC, Peiter PC, de Matos VP (2019). Spatial spread of malaria and economic frontier expansion in the Brazilian Amazon. PLoS ONE.

[4] Canelas T, Castillo-Salgado C, Ribeiro H (2018). Analyzing the local epidemiological profile of malaria transmission in the Brazilian Amazon Between 2010 and 2015. PLoS Curr..

[5] Oliveira-Ferreira J, Lacerda MVG, Brasil P, Ladislau JLB, Tauil PL, Daniel-Ribeiro CT (2010). Malaria in Brazil: an overview. Malar J.

[6] Siqueira AM, Bassat Q, Rodovalho S, Lacerda MVG (2017). Raising the red flag for malaria elimination and integrated fever surveillance in the Brazilian amazon. Lancet Glob Health.

[7] Braz RM, Tauil PL (2016). Santelli ACFES, Fontes CJF [Evaluation of the completeness and timeliness of malaria reporting in the Brazilian Amazon, 2003–2012]. Epidemiol Serv Saude.

[8] Wiefels A, Wolfarth-Couto B, Filizola N, Durieux L, Mangeas M (2016). Accuracy of the malaria epidemiological surveillance system data in the state of Amazonas. Acta Amazonica.

[9] Griffing SM, Tauil PL, Udhayakumar V, Silva-Flannery L (2015). A historical perspective on malaria control in Brazil. Mem Inst Oswaldo Cruz.

[10] Ferreira MU, Castro MC (2016). Challenges for malaria elimination in Brazil. Malar J.

[11] MacDonald AJ, Mordecai EA (2019). Amazon deforestation drives malaria transmission, and malaria burden reduces forest clearing. Proc Natl Acad Sci USA.

[12] de Castro MC, Monte-Mor RL, Sawyer DO, Singer BH (2006). Malaria risk on the Amazon frontier. Proc Natl Acad Sci USA.

[13] da Silva NS, da Silva-Nunes M, Malafronte RS, Menezes MJ, D'Arcadia RR, Komatsu NT (2010). Epidemiology and control of frontier malaria in Brazil: lessons from community-based studies in rural Amazonia. Trans R Sco Trop Med Hyg.

[14] Grillet ME, Hernández-Villena JV, Llewellyn MS, Paniz-Mondolfi AE, Tami A, Vincenti-Gonzalez MF (2019). Venezuela's humanitarian crisis, resurgence of vector-borne diseases, and implications for spillover in the region. Lancet Infect Dis.

[15] Rosas-Aguirre A, Guzman-Guzman M, Gamboa D, Chuquiyauri R, Ramirez R, Manrique PC (2017). Micro-heterogeneity of malaria transmission in the Peruvian Amazon: a baseline assessment underlying a population-based cohort study. Malar J..

[16] Cooper L, Kang SY, Bisanzio D, Maxwell K, Rodriguez-Barraquer I, Greenhouse B (2019). Pareto rules for malaria super-spreaders and super-spreading. Nat Commun.

[17] Instituto Brasileiro de Geografia e Estatística (IBGE). https://www.ibge.gov.br/. Accessed July 2020.

[18] Indicadores e Dados Básicos–IDB/SUS. www.datasus.gov.br. Accessed July 2020.

[19] Pfeffer DA, Lucas TCD, May D, Harris J, Rozier J, Twohig KA (2018). malariaAtlas: an R interface to global malariometric data hosted by the Malaria Atlas Project. Malar J.

[20] R Core Team (2018). R: a language and environment for statistical computing.

[21] Valle D, Lima JMT (2014). Large-scale drivers of malaria and priority areas for prevention and control in the Brazilian Amazon region using a novel multi-pathogen geospatial model. Malar J.

[22] dos Reis IC, Codeco CT, Degener CM, Keppeler EC, Muniz MM, de Oliveira FGS (2015). Contribution of fish farming ponds to the production of immature *Anopheles* spp. in a malaria-endemic Amazonian town. Malar J..

[23] Bejon P, Williams TN, Nyundo C, Hay SI, Benz D, Gething PW (2014). A micro-epidemiological analysis of febrile malaria in Coastal Kenya showing hotspots within hotspots. Elife.

[24] Almeida ACG, Kuehn A, Castro AJM, Vitor-Silva S, Figueiredo EFG, Brasil LW (2018). High proportions of asymptomatic and submicroscopic *Plasmodium vivax* infections in a peri-urban area of low transmission in the Brazilian Amazon. Parasit Vectors.

[25] Carneiro I, Roca-Feltrer A, Griffin JT, Smith L, Tanner M, Schellenberg JA (2010). Age-patterns of malaria vary with severity, transmission intensity and seasonality in sub-Saharan Africa: a systematic review and pooled analysis. PLoS ONE.

[26] Ladeia-Andrade S, Ferreira MU, de Carvalho ME, Curado I, Coura JR (2009). Age-dependent acquisition of protective immunity to malaria in riverine populations of the Amazon Basin of Brazil. Am J Trop Med Hyg.

[27] Magris M, Rubio-Palis Y, Alexander N, Ruiz B, Galvan N, Frias D, Blanco M, Lines J (2007). Community-randomized trial of lambdacyhalothrin-treated hammock nets for malaria control in Yanomami communities in the Amazon region of Venezuela. Trop Med Int Health.

[28] Castro MC, Baeza A, Codeço CT, Cucunubá ZM, Dal’Asta AP, De Leo GA (2019). Development, environmental degradation, and disease spread in the Brazilian Amazon. PLoS Biol..

[29] Alexander N, Rodriguez M, Perez L, Caicedo JC, Cruz J, Prieto G (2005). Case-control study of mosquito nets against malaria in the Amazon region of Colombia. Am J Trop Med Hyg.

[30] Graham M, Winter AK, Ferrari M, Grenfell B, Moss WJ, Azman AS (2019). Measles and the canonical path to elimination. Science.

[31] Almeida ED, Rodrigues LCS, Vieira JLF (2014). Estimates of adherence to treatment of vivax malaria. Malar J.

[32] Lacerda MVG, Bassat Q (2019). Primaquine for all: is it time to simplify malaria treatment in co-endemic areas?. Lancet Infect Dis.

[33] Siqueira AM, Mesones-Lapouble O, Marchesini P, Sampaio VS, Brasil P, Tauil PL (2016). *Plasmodium vivax* landscape in Brazil: scenario and challenges. Am J Trop Med Hyg.

[34] Thriemer K, Bobogare A, Ley B, Gudo CS, Alam MS, Anstey NM (2018). Quantifying primaquine effectiveness and improving adherence: a round table discussion of the APMEN Vivax Working Group. Malar J.

[35] Llanos-Cuentas A, Lacerda MVG, Hien TT, Vélez ID, Namaik-Larp C, Chu CS (2019). Tafenoquine versus primaquine to prevent relapse of *Plasmodium vivax* malaria. N Engl J Med.

[36] Lacerda MVG, Llanos-Cuentas A, Krudsood S, Lon C, Saunders DL, Mohammed R (2019). Single-dose tafenoquine to prevent relapse of *Plasmodium vivax* malaria. N Engl J Med.

[37] Hallal PC, Hartwig FP, Horta BL, Silveira MF, Struchiner CJ, Vidaletti LP (2020). SARS-CoV-2 antibody prevalence in Brazil: results from two successive nationwide serological household surveys. Lancet Glob Health.

[38] Rogerson SJ, Beeson JG, Laman M, Poespoprodjo JR, William T, Simpson JA (2020). Identifying and combating the impacts of COVID-19 on malaria. BMC Med.

