# Supplementary material for: The top 1%: quantifying the unequal distribution of malaria in Brazil
Source: Malar J. 2021 Feb 12;20:87. doi: 10.1186/s12936-021-03614-4 (PMC7880522; doi:10.1186/s12936-021-03614-4)
Supplement: Supplementary file 1 — Additional file 1: Table S1. Notified cases and incidence per 1000 population per year, malaria type, highest transmission state of the Amazon Basin. Figure S1. Monthly P. vivax and P. falciparum cases. Figure S2. P. vivax and P. falciparum 2018 cases stratified by municipality. Figure S3. States reporting cases imported from Venezuela. Figure S4. Proportion of P. vivax and P. falciparum cases by age group per year in each State stratified by gender. Figure S5. Proportion of cases in the top 1% of municipalities and health units. Figure S6. Gini coefficient and population size. [file 12936_2021_3614_MOESM1_ESM.docx]

**Supplementary Materials**

**Table S1: Notified cases and incidence per 1000 population per year, malaria type, highest transmission state of the Amazon Basin.** Mixed infections were double counted for *P. vivax* and *P. falciparum*. Here, notifications include all positive cases in SIVEP. The counts may not reflect the totals in some Figures, because these counts are obtained before data curation for the analysis, which effectively removed inconsistencies or notifications of cure verification thick smear.

| **Year** | ***P. vivax*** | | ***P. falciparum*** | | **All malaria** | | **Highest reporting state** | |
| --- | --- | --- | --- | --- | --- | --- | --- | --- |
|  | Cases | Incidence | Cases | Incidence | Cases | Incidence | Cases | Incidence |
| 2003 | 370,449 | 16.6 | 109,018 | 4.89 | 476,248 | 21.3 | Amazonas | Rondônia |
| 2004 | 421,621 | 18.2 | 140,616 | 6.08 | 557,454 | 24.1 | Amazonas | Rondônia |
| 2005 | 558,062 | 23.6 | 205,582 | 8.71 | 757,514 | 32.1 | Amazonas | Acre |
| 2006 | 511,957 | 21.3 | 189,857 | 7.89 | 694,866 | 28.9 | Amazonas | Acre |
| 2007 | 446,193 | 18.9 | 115,008 | 4.88 | 557,692 | 23.7 | Amazonas | Acre |
| 2008 | 324,821 | 13.3 | 57,723 | 2.37 | 380,287 | 15.6 | Amazonas | Amazonas |
| 2009 | 313,098 | 12.7 | 59,025 | 2.39 | 369,559 | 15.0 | Pará | Acre |
| 2010 | 358,508 | 14.1 | 58,610 | 2.30 | 414,081 | 16.3 | Pará | Roraima |
| 2011 | 281,412 | 10.9 | 37,560 | 1.45 | 316,894 | 12.3 | Pará | Acre |
| 2012 | 242,447 | 9.28 | 36,851 | 1.41 | 280,490 | 10.7 | Pará | Acre |
| 2013 | 171,159 | 6.34 | 32,438 | 1.20 | 205,018 | 7.60 | Amazonas | Acre |
| 2014 | 141,311 | 5.18 | 23,890 | 0.875 | 165,365 | 6.06 | Amazonas | Acre |
| 2015 | 147,542 | 5.34 | 16,645 | 0.602 | 165,511 | 5.99 | Amazonas | Acre |
| 2016 | 134,436 | 4.83 | 15,207 | 0.546 | 151,190 | 5.43 | Amazonas | Acre |
| 2017 | 214,036 | 7.57 | 21,362 | 0.755 | 237,955 | 8.41 | Amazonas | Acre |
| 2018 | 217,685 | 7.60 | 21,273 | 0.742 | 242,797 | 8.47 | Amazonas | Roraima |
| **Total** | **4,854,737**  **(81.3%)** | | **1,140,665**  **(19.1)%** | | **5,972,921** | | **Amazonas (34.8%)** | |


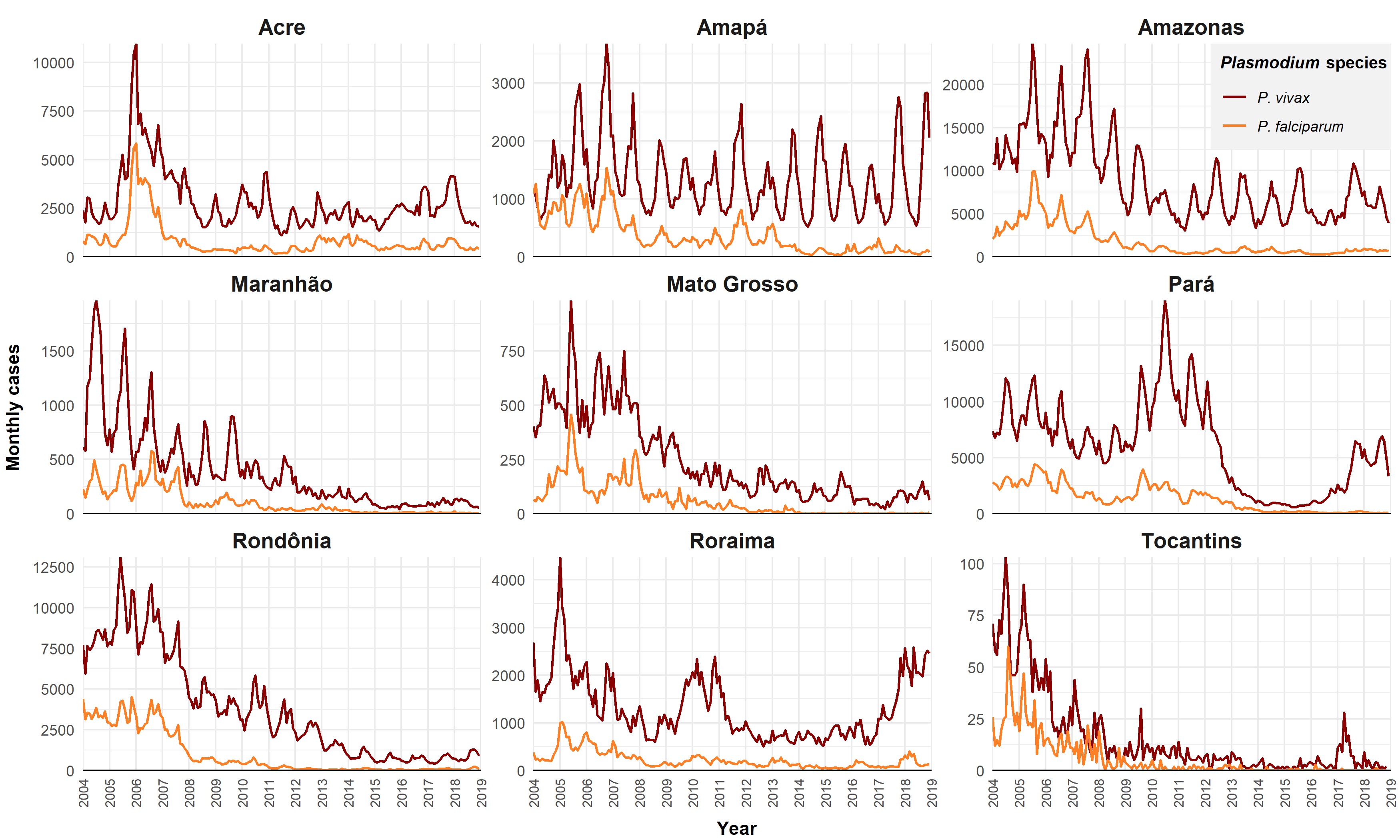
**Figure S1. Monthly *P. vivax* and *P. falciparum* cases.** The monthly time series span 2004 to 2018 and are stratified by the reporting malaria endemic state.


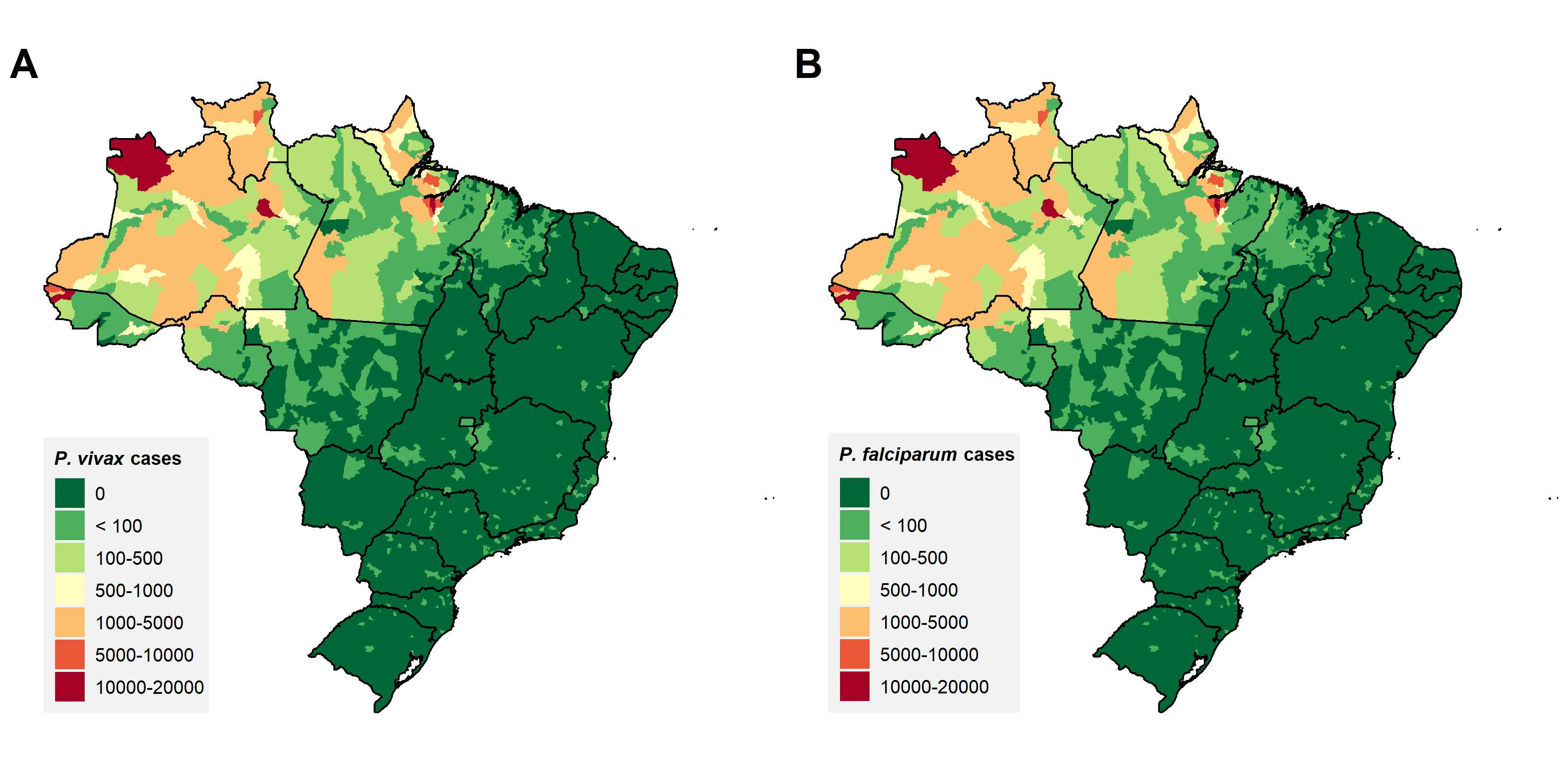
**Figure S2. *P. vivax* and *P. falciparum* 2018 cases stratified by municipality.**


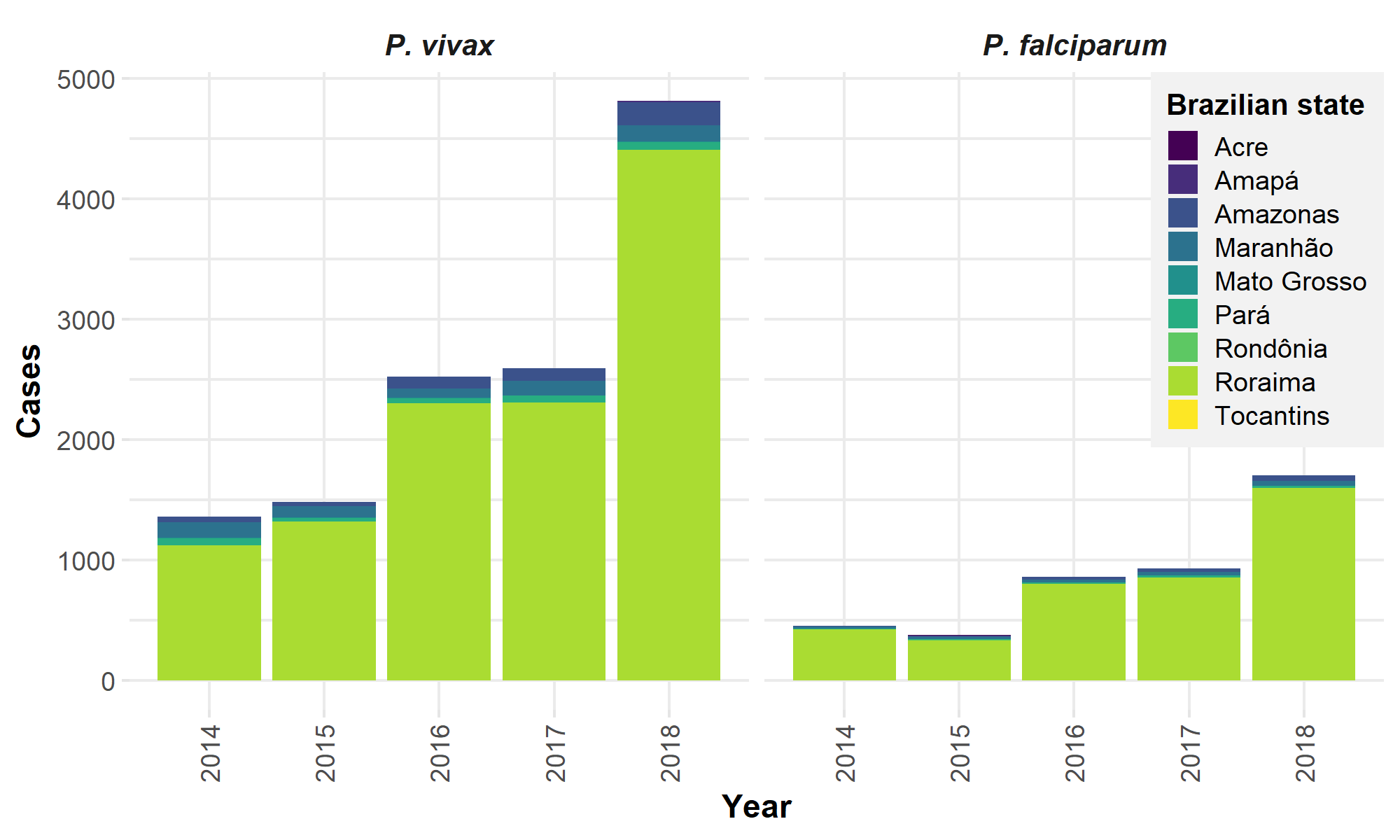
**Figure S3. States reporting cases imported from Venezuela.** Imported is defined as a country of probable infection other than Brazil. In the last 5 years, Brazil has seen a significant increase in the number of malaria cases originating from Venezuela potentially largely driven by the migrant population due to the humanitarian crisis. The health system in Roraima state border Venezuela has admitted the highest number of migrant cases in recent years.


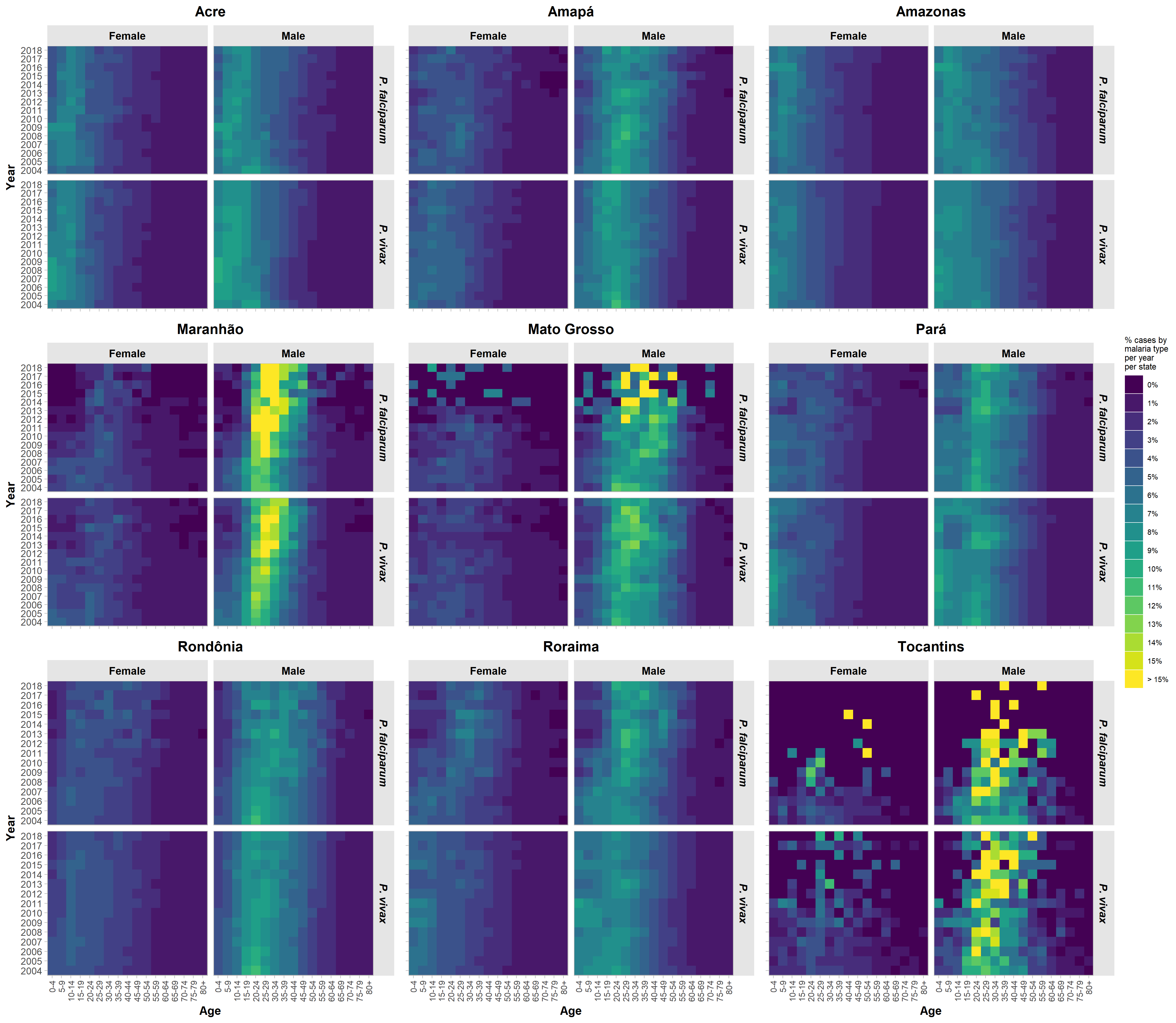
**Figure S4. Proportion of *P. vivax* and *P. falciparum* cases by age group per year in each state**

**stratified by gender.** Proportions were calculated for each gender with the denominator only considering the state, malaria type, and year. Note that for both *P. vivax* and *P. falciparum*, for a given year the total proportion of cases across all age groups in males and females sums to 1, i.e. the plots are normalised horizontally.


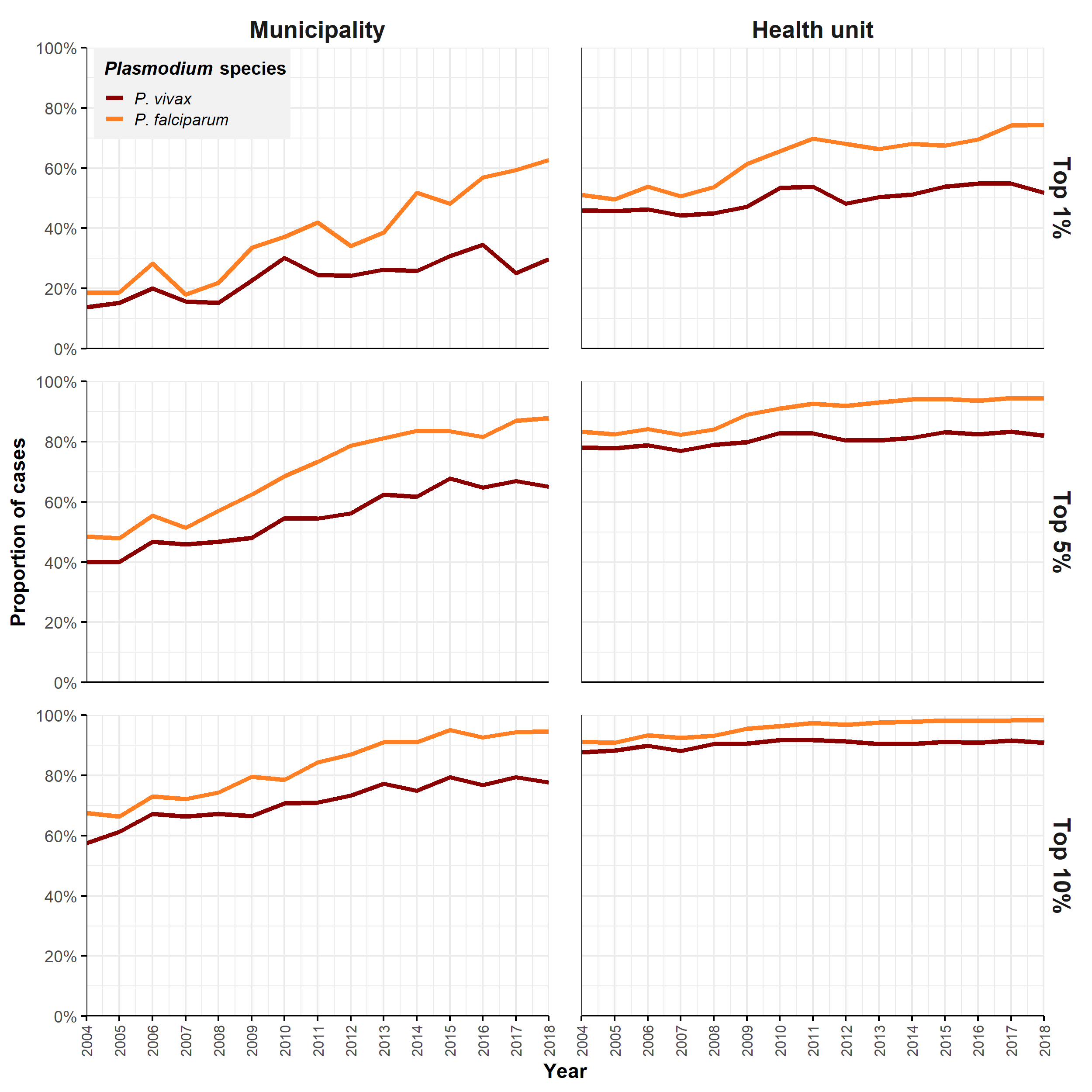
**Figure S5. Proportion of cases in the top 1% of municipalities and health units.** In each year, administrative units were ordered by cases numbers (*P. vivax* or *P. falciparum*). The proportion of total cases occurring in the top 1% of administrative units was then calculated. The same calculation was performed for the top 5% and 10% of administrative units.


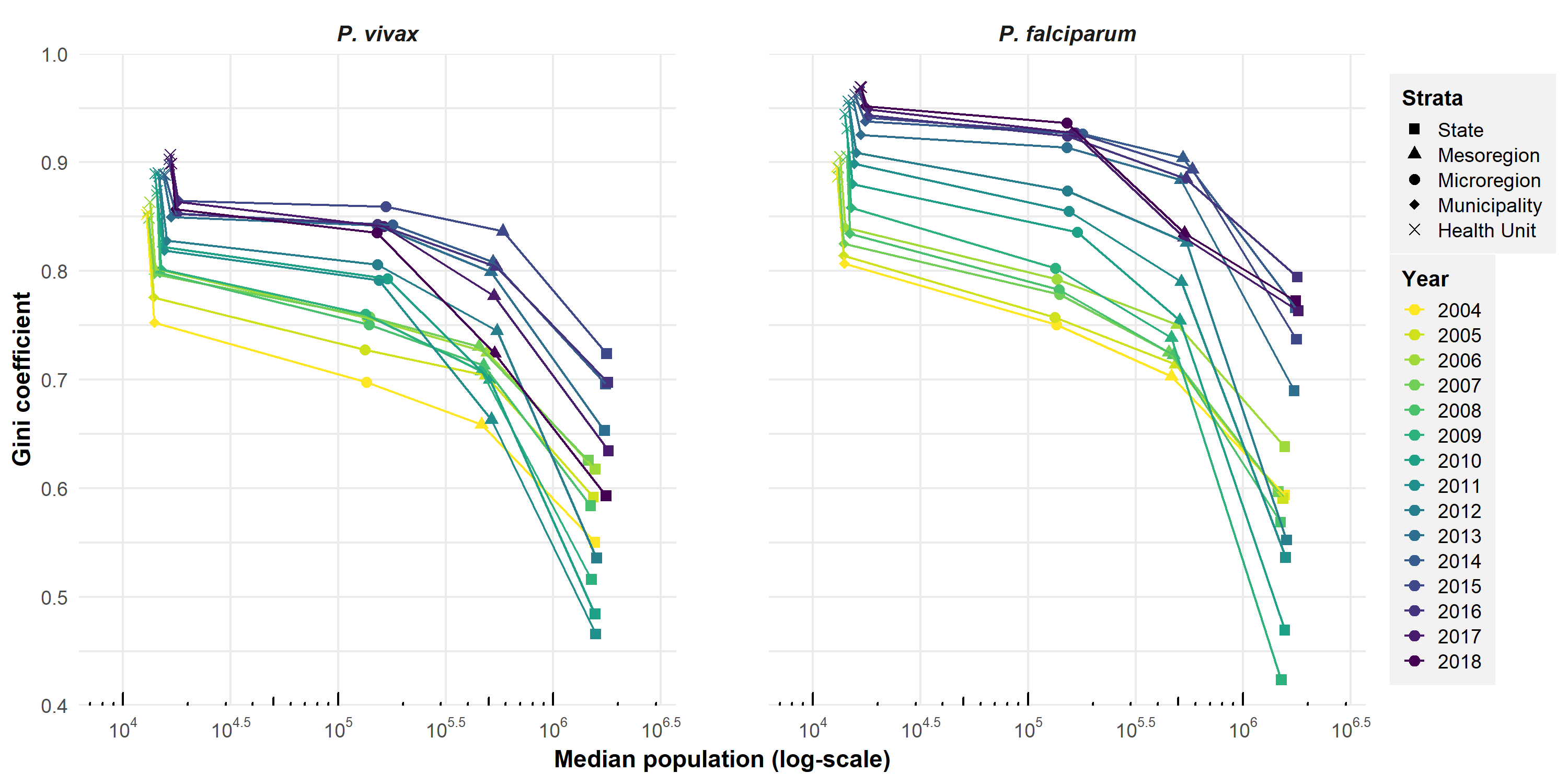
**Figure S6. Gini coefficient and population size.** Lower administrative strata correspond to smaller subpopulations. The Gini coefficient increases as the median subpopulation size decreases, consistent with measured heterogeneity being dependent on the level of population stratification used. As the population size increases, heterogeneity among strata becomes diluted, whereas in smaller populations variation becomes more apparent. Thus zooming into small populations allows better identification of different intensities of transmission pockets.
